# A Developed Jerk Sensor for Seismic Vibration Measurements: Modeling, Simulation and Experimental Verification

**DOI:** 10.3390/s23125730

**Published:** 2023-06-20

**Authors:** Mostafa M. Geriesh, Ahmed M. R. Fath El-Bab, Wael Khair-Eldeen, Hassan A. Mohamadien, Mohsen A. Hassan

**Affiliations:** 1 Material Science and Engineering Program, School of Innovative Design Engineering, Egypt-Japan University of Science and Technology (E-JUST), New Borg Al-Arab City 21934, Egypt; mohsen.khozami@ejust.edu.eg; 2 Civil Engineering Department, Faculty of Engineering, Suez Canal University, Ismailia 41522, Egypt; h_mohamadien@eng.suez.edu.eg; 3 Mechatronics and Robotics Department, School of Innovative Design Engineering, Egypt-Japan University of Science and Technology (E-JUST), New Borg Al-Arab City 21934, Egypt; ahmed.rashad@ejust.edu.eg; 4 Department of Industrial Engineering and Systems Management, School of Innovative Design Engineering, Egypt-Japan University of Science and Technology (E-JUST), New Borg Al-Arab City 21934, Egypt; wael.khaireldin@ejust.edu.eg

**Keywords:** jerk sensor, stainless steel cantilever, gyroscope, finite element modeling, seismic vibrations

## Abstract

Acceleration-based sensors are widely used in indicating the severity of damage caused to structural buildings during dynamic events. The force rate of change is of interest when investigating the effect of seismic waves on structural elements, and hence the calculation of the jerk is necessary. For most sensors, the technique used for measuring the jerk (m/s^3^) is based on differentiating the time–acceleration signal. However, this technique is prone to errors especially in small amplitude and low frequency signals, and is deemed not suitable when online feedback is required. Here, we show that direct measurement of the jerk can be achieved using a metal cantilever and a gyroscope. In addition, we focus on the development of the jerk sensor for seismic vibrations. The adopted methodology optimized the dimensions of an austenitic stainless steel cantilever and enhanced the performance in terms of sensitivity and the jerk measurable range. We found, after several analytical and FE analyses, that an L-35 cantilever model with dimensions 35 × 20 × 0.5 (mm^3^) and a natural frequency of 139 (Hz) has a remarkable performance for seismic measurements. Our theoretical and experimental results show that the L-35 jerk sensor has a constant sensitivity value of 0.05 ((deg/s)/(G/s)) with ±2% error in the seismic frequency bandwidth of 0.1~40 (Hz) and for amplitudes in between 0.1 and 2 (G). Furthermore, the theoretical and experimental calibration curves show linear trends with a high correlation factor of 0.99 and 0.98, respectively. These findings demonstrate the enhanced sensitivity of the jerk sensor, which surpasses previously reported sensitivities in the literature.

## 1. Introduction

During earthquakes, seismic waves in the form of an S-wave, P-wave, Rayleigh-wave and Love-wave propagate in all directions, causing the ground to vibrate with a frequency ranging from 0.1 to 40 (Hz) and peak ground acceleration ranging from 0.1 to 2 (G), which depend strongly on the magnitude and hypocentral distance [1,2]. As a result, damage to buildings occurs if they cannot sustain these vibrations. In addition, seismic vibrations of low frequency and small amplitudes may cause severe cumulative damage with fatigue to structural elements. It is important for health monitoring systems of structural buildings and bridges to develop a new technique for directly measuring the jerk and the rate of change of force during seismic vibrations. The development of such sensors is also needed in many other applications such as elevators, robots, automotive safety, crashworthiness, material fatigue, aerospace and ballistic testing [3].

Generally, seismometers are used to detect seismic waves. A simple seismometer consists of a mass attached to a fixed frame. The relative motion between the mass and the frame provides a measurement of the vertical ground motion. The modern seismometers are driven by force-balanced complex electronic controllers that hold the small mass stationary during the ground movement through a feedback control circuit. The force necessary to hold the mass motionless relative to the frame is proportional to the acceleration [4]. Geophones are also used to detect seismic waves in terms of ground motion. Most of the currently available geophones consist of a mass suspended by mechanical springs. Mass movement is controlled by either magnets or coils. The response of a coil/magnet geophone is proportional to the ground velocity. However, the mechanical spring decreases the device’s performance, and the geophone’s sensitivity to ground motion is variable with input frequency [5]. Therefore, modern seismometers and geophones measure acceleration instead of the velocity of ground movement. In other words, modern types are acceleration-based sensors.

Accelerometer sensors measure acceleration and indirectly detect the input jerk in dynamic systems [6,7]. Accelerometer sensors must have high responsiveness and resolution to sense acceleration accurately, and this is achieved by maintaining a high Q-factor to prevent any reduction in the sensitivity due to increased damping [8,9]. Unfortunately, the current measurement of the jerk using accelerometers is mainly based on signal processing by differentiating the input acceleration with respect to time [10,11,12,13,14,15,16,17,18,19]. This indirect technique of measuring the input jerk consumes more calculations and time. In addition, high error levels due to noise sensitivity and small-time steps are expected. This technique may lead to imprecise calculations of the jerk value in small amplitude and low frequency signals, especially in health monitoring systems of structural buildings [9]. Consequently, many attempts have been made to overcome this problem; Rangel-Magdaleno et al. [20] developed a sensor for jerk monitoring with finite differences of the acceleration signal. They used a standard accelerometer with an improved signal-to-quantization noise ratio. Their idea is based on improved oversampling techniques that give a better estimation of the jerk than that produced by a Nyquist-rate differentiator. Xueshan et al. [21] proposed a different approach to measure the input jerk signal. Their idea is based on absolute motion detection using a mass-spring system with an electromechanical coupling circuit to substitute traditional differential circuits. They developed four models called JW-1, JW-2, JW-3 and JW2-3D. However, in these models, they neglected the damping coefficient, and so the sensor sensitivity decreased with increasing the excitation frequency. For instance, the JW-1 model sensitivity was 0.08 (mV/(m/s^3^)) for a bandwidth of 0~100 (Hz). Kubota et al. [22] developed a servo-type jerk sensor for an air-type anti-vibration system. This type of servo jerk sensor is mainly based on integral feedback control. They improved their jerk sensor’s phase and sensitivity characteristics by increasing the stiffness of the plate spring of the pendulum in the sensor. Manabe et al. [23,24] proposed a servo-type jerk sensor based on controlling the zero position of a pendulum through velocity feedback control (i.e., PI compensator), which auto corrects the position, enhances stability and reduces oscillation. Li et al. [25] proposed a direct method for measuring the jerk using a fiber optic jerk sensor (FOJS) based on a differentiating Mach–Zehnder interferometer. If the sensing probe detects a jerk signal, the fiber winding on the probe stretches and modulates the phase of the interferometer. Thus, the jerk is measured by measuring the absolute phase of the interference light. In 1999, Tamura et al. [26] proposed a direct method for sensing the jerk with a cantilever-based jerk sensor. They measured the output voltage of a vibratory gyroscope mounted on an aluminum cantilever to detect discontinuities in the response of buildings under earthquakes based on the continuous vibration system theory. They reported two sensor models named A and B. However, the model with the higher sensitivity has significantly large dimensions of 145 × 30 × 5 (mm^3^) and a low sensitivity of 0.01 ((deg/s)/(G/s)). However, in practical applications, lower dimensions and a higher sensitivity are needed. Therefore, more research is required to enhance the sensitivity of the cantilever-based jerk sensor proposed by Tamura [26].

In earthquake engineering, jerk sensors are not common. Consequently, several methods have been used to try to quantify the jerk during seismic vibrations [27]. Tong et al. [28] evaluated the basic characteristics (amplitude, duration and frequency component) of the time derivative of earthquake acceleration (TDoA) based on records from the 1999 Chi-Chi earthquake (Mw 7.6) and one of its aftershocks (Mw 6.2). Using a mid-point differentiation formula, they extracted the TDoA time series with direct numerical differentiation of the ground acceleration time series. They found that the maximum TDoA recorded at a free-field station was over 31.8 (G/s), with a total error within 4%. They stated that, in the case of large TDoA, the structural response might be accompanied by stress concentration and local damages due to inhomogeneous dynamic loading resulting from the stress wave propagation. Haoxiang et al. [7] established a numerical computing method for measuring elastic and inelastic jerk response spectra of structures under seismic activity. They calculated the jerk spectrum directly by establishing state–space equations and using the fourth-order Runge–Kutta method for solving the structural acceleration response. The jerk response is obtained by calculating the time derivation of acceleration. The results showed that the jerk spectrum had similar rules to the acceleration spectrum in general, and the amplitude was relative to the predominant period, especially for structures with a short or medium period. Other researchers used damage detection methods based on the jerk energy of the structural response; An et al. [19] used a jerk energy-based damage index in defining two damage localization procedures, namely, mean normalized curvature difference of waveform jerk energy and curvature difference probability waveform jerk energy. After experimentation on a six-story lumped-mass shear building model, they found that these methods have much better accuracy in determining localized damage and a better performance than model-based methods in the presence of noise. However, these methods are classified as model-free in which damage detection is based on the vibration response time series signals rather than numerical models.

From previous studies, the detection of the input jerk during seismic vibration is commonly based on the differentiation of the measured acceleration. However, very few works have been conducted to measure the input jerk directly. Therefore, this study focuses on enhancing the sensitivity of a cantilever-based sensor for direct measurement of the jerk during seismic events. The value of the enhanced jerk sensor lies in providing high sensitivity, reducing signal processing time and eliminating differentiation errors, which are necessary for small amplitude and low frequency signals. In other words, the enhanced sensor provides online jerk measurement without performing signal differentiation in contrast to geophones, accelerometers and seismometers. The enhanced jerk sensor was modeled, designed and simulated using analytical equations and ANSYS 2020R2 finite element analysis software to obtain the sensitivity frequency response and theoretical calibration curves. The enhanced design of the cantilever-based jerk sensor is based on optimizing the dimensions of an austenitic spring stainless steel 304 metallic cantilever to increase the sensitivity. The sensor dimensions were chosen based on the excitation frequency range of seismic vibrations. The best sensor model design has been fabricated and experimentally tested on a high-excitation computer-controlled shaker. The rest of the paper is organized into three main sections as follows: Section 2 (includes the sensor structure, sensor analytical model, sensor design, sensor FE modeling and simulation work and experimental work), Section 3 (includes FE results, experimental verification and performance comparison) and finally, Section 4 (summary and conclusions).

## 2. Materials and Methods

### 2.1. Sensor Structure

**Figure 1:**
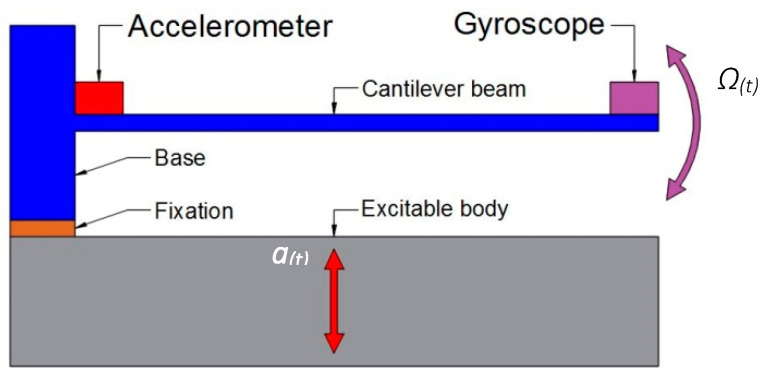
A schematic diagram for the components of the jerk sensor.

The jerk sensor consisted of a metal cantilever with a gyroscope mounted at its free tip, and an accelerometer mounted at its fixed end, as shown in Figure 1. The accelerometer was used once in practice to calibrate the jerk sensor sensitivity. When the jerk sensor (cantilever and gyroscope) was subjected to an excitation signal ($a_{\left(t\right)}$), the jerk amplitude ($j_{\left(t\right)}$) was determined from the gyroscope reading (Ω) (angular velocity) and jerk sensitivity ($S_{j}$), which will be explained in the next section.

### 2.2. Sensor Analytical Model

The jerk ($j_{\left(t\right)}$) is a physical signal that describes the rate of change of acceleration ($a_{\left(t\right)}$), or in other words, it is the first derivative of acceleration with respect to time ($j_{\left(t\right)}=da_{\left(t\right)}/dt$), and its unit is m/s^3^. The rate of change of force can be related to the jerk when mass is constant by differentiating Newton’s second law ($F_{\left(t\right)}=ma_{\left(t\right)}$) with respect to time as described in Equation (1).

(1) $$\frac{dF_{\left(t\right)}}{dt}=m\frac{da_{\left(t\right)}}{dt}=mJ_{\left(t\right)}$$

**Figure 2:**
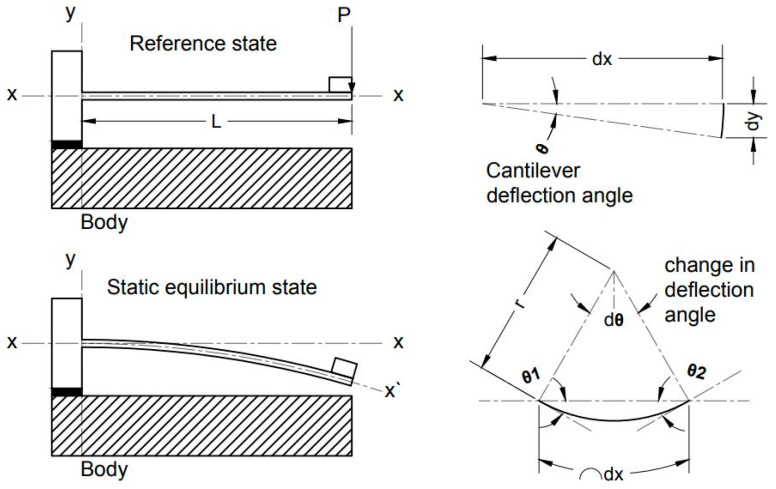
Schematic diagram showing the change in deflection angle along the length of a cantilever beam.

Consider a cantilever beam as shown in Figure 2; when the beam is statically analyzed under constant acceleration (a=gravitational acceleration), the beam self-mass and the attached mass at the cantilever tip induce a static force that causes a static deflection (dy).

From solid mechanics [29], as the moment (M) on the beam increases, the radius of curvature (r) decreases; thus, the relationship is inversely proportional. The constant can be determined based on the geometry of the cross-section (I) and the beam’s material stiffness (E):

(2) $$M_{1}r_{1}=M_{2}r_{2}=const.=EI\;\frac{1}{r}=\frac{M}{EI}$$

where $M_{1}$ and $M_{2}$ are arbitrary moment values causing curvature radii ($r_{1}$) and ($r_{2}$), respectively. By referring to Figure 2, the change in the slope of deflection is dθ=dx/r; thus, the slope of deflection ($\theta_{\left(x\right)}$) can be related to the moment from Equation (2):

(3) $$\theta_{\left(x\right)}=\int \frac{1}{r}dx=\int \frac{M_{\left(x\right)}}{EI}dx$$

Therefore, by using Equation (3), the deflection ($y_{\left(x\right)}$) can be obtained:

(4) $$\frac{dy_{\left(x\right)}}{dx}=\tan\theta_{\left(x\right)}\approx \theta_{\left(x\right)}[rad]\;y_{\left(x\right)}=\int \theta_{\left(x\right)}dx=\int \int \frac{M_{\left(x\right)}}{EI}{dx}^{2}$$

where $M_{\left(x\right)}$ is the total moment as a function of x and can be calculated using the superposition method by summing up the moment from the gyroscope mass ($M_{1\left(x\right)}$) and the moment from the cantilever self-mass ($M_{2\left(x\right)}$). Assuming a constant acceleration (a) is acting on the masses perpendicular to the *x*-axis, the resulting moment ($M_{\left(x\right)}$) can be calculated as:

$$M_{\left(x\right)}=M_{1(x)}+M_{2(x)}$$

For the moment resulting from the gyroscope mass ($M_{1(x)}$):

$$M_{1(x)}=-m_{1}aL+m_{1}ax\;M_{1(x)}=m_{1}a(x-L)$$

Thus, the angle of deflection due to the gyroscope mass ($\theta_{1(x)}$) can be calculated as the first integral with respect to dx:

(5) $$\theta_{1(x)}=\int \frac{M_{1(x)}}{EI}dx=\frac{m_{1}a}{EI}\int \left(x-L\right)dx\;\theta_{1(x)}=\frac{m_{1}a}{EI}\left(\frac{x^{2}}{2}-Lx+c_{1}\right)$$

Note that at x=0, due to fixed support, $\theta_{1(x)}=0$; therefore, $c_{1}=0$. For the moment resulting from the cantilever self-mass ($M_{2(x)}$):

$$M_{2\left(x\right)}=\frac{-m_{2}aL^{2}}{2}+m_{2}aLx-\frac{m_{2}ax^{2}}{2}\;M_{2\left(x\right)}=-\frac{m_{2}a}{2}(x^{2}-2Lx+L^{2})$$

Thus, the angle of deflection due to the cantilever self-mass ($\theta_{2(x)}$) can be calculated as the first integral with respect to dx:

(6) $$\theta_{2(x)}=\int \frac{M_{2(x)}}{EI}dx=-\frac{m_{2}a}{2EI}\int \left(x^{2}-2Lx+L^{2}\right)dx\;\theta_{2(x)}=-\frac{m_{2}a}{2EI}\left(\frac{x^{3}}{3}-Lx^{2}+L^{2}x+c_{2}\right)$$

Note that at x=0, due to fixed support, $\theta_{2(x)}=0$; therefore, $c_{2}=0$. Therefore, the total cumulative angle of deflection ($\theta_{\left(x\right)}$) measured from the fixed end towards any position along the cantilever length is given as the sum of Equations (5) and (6):

(7) $$\theta_{\left(x\right)}=\theta_{1(x)}+\theta_{2(x)}\;\theta_{\left(x\right)}=\frac{m_{1}a}{EI}\left(\frac{x^{2}}{2}-Lx\right)-\frac{m_{2}a}{2EI}\left(\frac{x^{3}}{3}-Lx^{2}+L^{2}x\right)\;\theta_{\left(x\right)}=\frac{a}{EI}\left(m_{1}\left(\frac{x^{2}}{2}-Lx\right)-\frac{m_{2}}{2}\left(\frac{x^{3}}{3}-Lx^{2}+L^{2}x\right)\right)$$

From Equation (4), the deflection ($y_{\left(x\right)}$) at any position can be calculated by integrating the total angle of deflection ($\theta_{\left(x\right)}$) obtained from Equation (7):

(8) $$y_{\left(x\right)}=\int \theta_{\left(x\right)}dx=\frac{a}{EI}\int \left(m_{1}\left(\frac{x^{2}}{2}-Lx\right)-\frac{m_{2}}{2}\left(\frac{x^{3}}{3}-Lx^{2}+L^{2}x\right)\right)dx\;y_{\left(x\right)}=\frac{a}{EI}\left(m_{1}\left(\frac{x^{3}}{6}-\frac{Lx^{2}}{2}\right)-\frac{m_{2}}{2}\left(\frac{x^{4}}{12}-\frac{{Lx}^{3}}{3}+\frac{L^{2}x^{2}}{2}\right)+c_{3}\right)$$

Note that at x=0, due to fixed support, y=0; therefore, $c_{3}=0$. Therefore, the angle of deflection and the deflection at the cantilever tip (x=L) can be evaluated:

(9) $$\theta_{tip}=\theta_{\left(x=L\right)}=-\frac{L^{2}}{EI}\left(\frac{m_{1}}{2}+\frac{{\overset{-}{m}}_{2}L}{6}\right)a$$

(10) $$y_{tip}=y_{\left(x=L\right)}=-\frac{L^{3}}{EI}\left(\frac{m_{1}}{3}+\frac{{\overset{-}{m}}_{2}L}{8}\right)a=-\delta_{tip}.a$$

Then, $\delta_{tip}$ becomes the deflection due to a unit acceleration. So, the spring stiffness (k) (the force required to generate a unit deflection) at the cantilever tip can be calculated:

(11) $$y_{tip}=\frac{F_{tip}}{k_{tip}}=\frac{m_{total}.a}{k_{tip}}=\delta_{tip}[\frac{mm}{mm/s^{2}}].a[\frac{mm}{s^{2}}]$$

(12) $$\frac{m_{total}}{k_{tip}}=\delta_{tip}=\frac{m_{1}}{\frac{3EI}{L^{3}}}+\frac{{\overset{-}{m}}_{2}L}{\frac{8EI}{L^{3}}}$$

**Figure 3:**
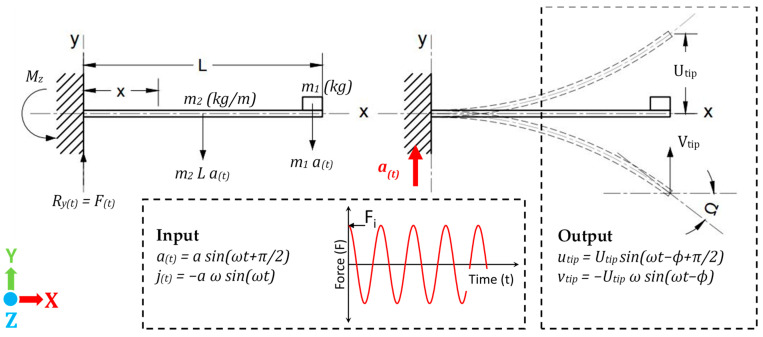
Schematic diagram showing dynamic oscillation of the cantilever beam due to input excitation.

When the cantilever is subjected to harmonic forced vibrations, e.g., earthquake waves, the cantilever tip will oscillate at the input frequency as an underdamped single degree of freedom (SDOF) system subjected to base excitation, as shown in Figure 3. A harmonic force ($f_{\left(t\right)}$) is applied on the system due to the dynamic acceleration ($a_{\left(t\right)}$) acting upon the masses $m_{1}$ and ${\overset{-}{m}}_{2}$. A dynamic deflection wave ($u_{\left(x,t\right)}$) is generated having the same frequency ($\omega_{i}$) as the input acceleration, but due to response lagging, there will be a phase shift (∅) between $u_{\left(x,t\right)}$ and $f_{\left(t\right)}$ [30].

The cantilever equation of motion can be represented as:

(13) $$m\ddot{u}+c\dot{u}+ku=-f_{\left(t\right)}$$

(14) $$f_{\left(t\right)}=ma_{\left(t\right)}=ma_{i}\sin\left(\omega_{i}t+\frac{\pi }{2}\right)$$

where c is the damping coefficient, k is the cantilever spring constant and m is the effective mass of the system, which equals $m_{1}+0.25{\overset{-}{m}}_{2}L$. The solution of Equation (13) [31] is the sum of the homogeneous ($u_{\left(x,t\right)h}$) and particular ($u_{\left(x,t\right)p}$) solutions given as:

$$u_{\left(x,t\right)}=u_{\left(x,t\right)h}+u_{\left(x,t\right)p}=U_{0(x)}e^{-\zeta \omega_{n}t}\sin\sqrt{1-\zeta^{2}}\omega_{n}t+U_{1(x)}\sin\left(\omega_{i}t-\phi +\frac{\pi }{2}\right)$$

The homogenous solution diminishes with time; therefore, only the particular solution of the function is considered because it represents the solution due to the effect of the input signal when the system reaches dynamic stability after passing the transient time. The deflection ($u_{\left(x,t\right)}$) of the underdamped vibration is given by:

(15) $$u_{\left(x,t\right)}=U_{1(x)}\sin\left(\omega_{i}t-\phi +\frac{\pi }{2}\right)$$

The amplitude ($U_{tip}$) and phase angle (ϕ) at the tip in Equation (15) are given by:

$$U_{tip}=\frac{{\left(F_{i}/k\right)}_{tip}}{\sqrt{{\left(2\zeta \beta \right)}^{2}+{\left(1-\beta^{2}\right)}^{2}}}\;\phi =\tan^{-1}\frac{2\zeta \beta }{1-\beta^{2}}$$

where β is the ratio of the excitation frequency ($\omega_{i}$) to the natural frequency ($\omega_{n}$). At initial condition (t=0), the cantilever is still in the static state with zero frequency ($\omega_{i}=0$) and β=0, thus the deflection at the cantilever tip ($u_{\left(x=L,t=0\right)}$) is equal to the static deflection ($y_{tip}$) of Equation (10), and since the motion of the cantilever tip is represented by SDOF, then:

(16) $$u_{\left(x=L,t=0\right)}=U_{tip}={\left(F/k\right)}_{tip}=y_{tip}$$

Therefore, from Equation (16), the amplitude ($U_{tip}$) and the dynamic deflection ($u_{\left(x=L,t\right)}$) at the cantilever tip at any t and β value are:

(17) $$U_{tip}=\frac{y_{tip}}{\sqrt{{\left(2\zeta \beta \right)}^{2}+{\left(1-\beta^{2}\right)}^{2}}}\;u_{\left(x=L,t\right)}=\frac{y_{tip}}{\sqrt{{\left(2\zeta \beta \right)}^{2}+{\left(1-\beta^{2}\right)}^{2}}}\sin\left(\omega_{i}t-\phi +\frac{\pi }{2}\right)$$

The dynamic angle of deflection ($\theta_{\left(x=L,t\right)}$) can be obtained by differentiating the dynamic deflection function with respect to dx:

(18) $$\theta_{\left(x=L,t\right)}={\left.\frac{du_{\left(x,t\right)}}{dx}\right|}_{x=L}=\frac{\theta_{tip}}{\sqrt{{\left(2\zeta \beta \right)}^{2}+{\left(1-\beta^{2}\right)}^{2}}}\sin\left(\omega_{i}t-\phi +\frac{\pi }{2}\right)$$

The angular velocity ($\Omega_{\left(x=L,t\right)}$) is the rate of change of the angle of deflection ($\theta_{\left(x=L,t\right)}$) and can be calculated by taking its first derivative with respect to time (dt) (it shows how quickly the angle changes with time and corresponds to the gyroscope readings):

(19) $$\Omega_{\left(x=L,t\right)}=\frac{d\theta_{\left(x=L,t\right)}}{dt}=-\frac{\theta_{tip}}{\sqrt{{\left(2\zeta \beta \right)}^{2}+{\left(1-\beta^{2}\right)}^{2}}}\omega_{i}\sin\left(\omega_{i}t-\phi \right)$$

The denominator is considered as a dynamic amplification factor, so the angular velocity ($\Omega_{\left(x=L,t\right)}$) can be calculated using Equations (7) and (19):

(20) $$\Omega_{\left(x=L,t\right)}=\frac{\frac{L^{2}}{EI}\left(\frac{m_{1}}{2}+\frac{{\overset{-}{m}}_{2}L}{6}\right)}{\sqrt{{\left(2\zeta \beta \right)}^{2}+{\left(1-\beta^{2}\right)}^{2}}}\omega_{i}a_{i}\sin\left(\omega_{i}t-\phi \right)$$

**Figure 4:**
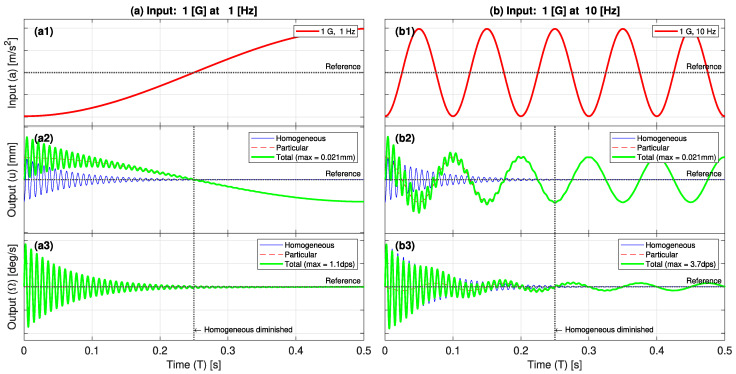

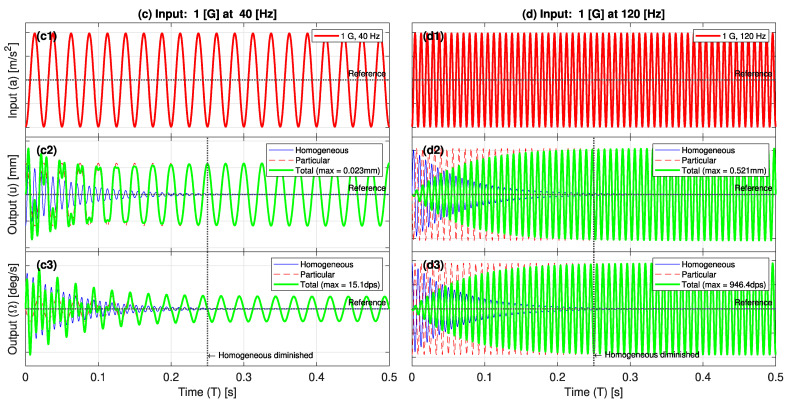
Graphical representations of the input acceleration and the output deflection and angular velocity responses at the cantilever tip (**a**) at 1 (G) and 1 (Hz); (**b**) at 1 (G) and 10 (Hz); (**c**) at 1 (G) and 40 (Hz); and (**d**) at 1 (G) and 120 (Hz).

Figure 4 shows graphical representations of the input acceleration (Equation (14)) as well as the output deflection (Equation (17)) and angular velocity (Equation (20)) responses at the cantilever tip at input frequencies of 1, 10, 40 and 120 (Hz) and an acceleration of 1 (G) for a short period of 0.5 (s). Representations are constructed considering a cantilever of a natural frequency of 120 (Hz) (3 times the seismic frequency of 0.1~40 (Hz)) and arbitrary geometrical and material parameters. In all cases, the effect of the homogeneous solution diminishes after a very short period, hence the neglection of the homogeneous solution. In addition, at the same input acceleration (acceleration acts as a scaling factor) as the input frequency increases, the increase in the deflection amplitude is small. However, the increase in the angular velocity is more significant, hence the suitability of the gyroscope in sensing the input.

Equation (20) represents the gyroscope reading at the cantilever tip, and this value was utilized to measure the sensitivity of the jerk sensor ($S_{a}$) to the input acceleration ($a_{\left(t\right)}$) and, similarly, the jerk sensor sensitivity ($S_{j}$) to the input jerk ($j_{\left(t\right)}$), as given in Equations (21) and (23), respectively.

(21) $$S_{a}=\frac{{\left|\Omega_{\left(L,t\right)}\right|}_{max}}{{\left|a_{\left(t\right)}\right|}_{max}}=\frac{\frac{L^{2}}{EI}\left(\frac{m_{1}}{2}+\frac{{\overset{-}{m}}_{2}L}{6}\right)\omega_{i}}{\sqrt{{\left(2\zeta \beta \right)}^{2}+{\left(1-\beta^{2}\right)}^{2}}}$$

(22) $$j_{\left(t\right)}=\left|-j\omega_{i}a_{\left(t\right)}\right|=\omega_{i}a_{i}\cos\left(\omega_{i}t+\frac{\pi }{2}\right)=-\omega_{i}a_{i}\sin\left(\omega_{i}t\right)$$

(23) $$S_{j}=\frac{{\left|\Omega_{\left(L,t\right)}\right|}_{max}}{{\left|j_{\left(t\right)}\right|}_{max}}=\frac{\frac{L^{2}}{EI}\left(\frac{m_{1}}{2}+\frac{{\overset{-}{m}}_{2}L}{6}\right)}{\sqrt{{\left(2\zeta \beta \right)}^{2}+{\left(1-\beta^{2}\right)}^{2}}}$$

From Equation (21), the jerk sensor sensitivity ($S_{a}$) to input acceleration ($a_{\left(t\right)}$) does not depend on the amplitude of the input acceleration; therefore, $S_{a}$ is a constant value for all acceleration amplitudes of the same frequency. However, $S_{a}$ is significantly affected by any variation in the excitation angular frequency ($\omega_{i}$). Nevertheless, the variation is deemed linear at small (β) ratios less than 0.3~0.2. Similarly, Equation (23) shows that the jerk sensor sensitivity ($S_{j}$) to the input jerk ($j_{\left(t\right)}$) does not depend on the amplitude of the input acceleration. In addition, $\omega_{i}$ is cancelled from the numerator; therefore, $S_{j}$ is only affected by the variation of $\omega_{i}$ that is introduced through $\beta =\omega_{i}/\omega_{n}$. Consequently, the effect of variation of $\omega_{i}$ on $S_{j}$ can be further reduced by controlling β. In other words, controlling the cantilever’s natural frequency ($\omega_{n}$) to be 3~5 times the measurable excitation frequency range enables us to design a jerk sensor with an almost constant $S_{j}$, which will be demonstrated in Section 3. Equations (21)–(23) also give the theoretical calibration of the jerk sensor sensitivity as follows:

(24) $$S_{j}=\frac{S_{a}}{\omega_{i}}$$

(25) $$j_{\left(t\right)}=\frac{\left|\Omega \right|}{S_{j}}=\frac{|\Omega |\times \omega_{i}}{S_{a}}$$

It is clear from Equations (24) and (25) that $S_{j}$ is less than $S_{a}$, and the estimated jerk ($j_{\left(t\right)}$) is greater than the input acceleration ($a_{\left(t\right)}$). Therefore, this sensor is very efficient in sensing any variation in the input signal with a small amplitude. Regarding Section 2.5, an accelerometer was used once to determine the calibration sensitivity curves $S_{a}$ and $S_{j}$. After that, the jerk value was obtained with direct measurement from the angular velocity sensor only. There is no need to use an accelerometer or any differentiation of the acceleration signal with respect to time.

### 2.3. Sensor Design

It is known that the natural frequency of the cantilever is a function of its geometry, material density and elastic modulus, as expressed in Equation (26). The desired dimensions and natural frequency ($f_{n}$) of the cantilever were designed to have a natural frequency 3~5 times the seismic vibration range (0.1~40 (Hz)) [32,33,34], which provides good-quality earthquake measurements. In other words, the excitation to the natural frequency ratio β, in Equation (23), was 0.33~0.20, which ensures $S_{j}$ to be constant over the seismic vibration range. It is worth mentioning that the frequency range 0.1~40 (Hz) also covers the ASTM-E606 low-cyclic fatigue test.

(26) $$2\pi f_{n}=\sqrt{\frac{k}{m}}≅\sqrt{\frac{\frac{k_{1}+k_{2}}{2}}{m_{1}+{\overset{-}{m}}_{2}L}}$$

where $k_{1}=3EI/L^{3}$ is the cantilever stiffness due to the gyroscope point mass ($m_{1}$) and $k_{2}=8EI/L^{3}$ is the cantilever stiffness due to its distributed self-mass (${\overset{-}{m}}_{2}$); stiffness is derived from Equation (12).

To study the effect of cantilever dimensions on the first mode natural frequency, 18 dimension sets were studied to estimate the first mode natural frequencies ($f_{n}$) using Equation (26). The first mode natural frequency was also estimated from an FE modal analysis for the 18 cantilever dimension sets. Table 1 summarizes the estimated natural frequencies of the 18 cantilever dimension sets as obtained from Equation (26) and FEA. The 10th dimension set denoted by L-35 was selected since it satisfied the recommended design criteria.

### 2.4. Sensor FE Modeling and Simulation Work

**Figure 5:**
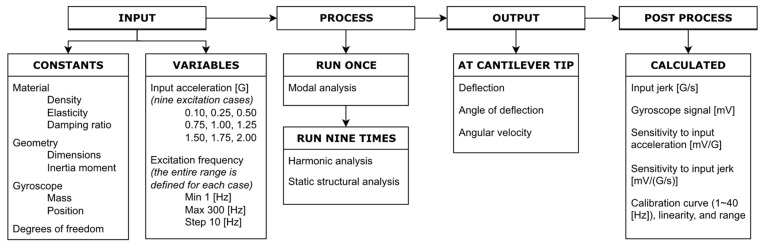
Flow chart of the simulation work.

The L-35 jerk sensor simulation model was constructed and analyzed using ANSYS 2020R2 FE software. Modal and Harmonic analyses were performed to obtain the theoretical sensitivities and calibration curves. A flow chart of the simulation work is illustrated in Figure 5.

#### 2.4.1. Material Properties

The stainless steel mechanical properties of the L-35 cantilever model are listed in Table 2. Among these properties, Young’s modulus, density and damping ratio were the most important properties affecting the natural frequency of the cantilever. For this purpose, the material chosen was high resilience spring stainless steel to store more elastic energy and reduce the energy loss due to vibration as much as possible.

#### 2.4.2. Geometrical Model

**Figure 6:**
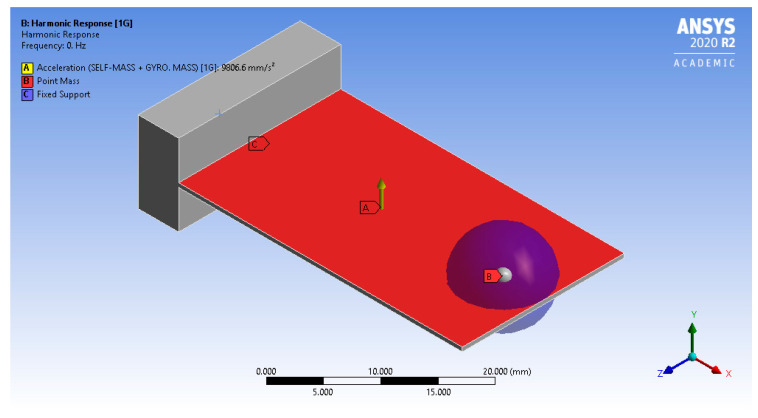
L-35 cantilever geometrical model and boundary conditions.

Based on the design criteria, the L-35 cantilever geometrical model of dimensions 35 × 20 × 0.5 (mm^3^) was constructed and attached to a fixed base of dimensions 5 × 20 × 10 (mm^3^), as shown in Figure 6. The printed circuit board (PCB) size for the suggested MPU9250 gyroscope mounted on the cantilever free tip was 25.5 × 15.4 × 1.5 (mm^3^). This size was considered when representing the affecting area of the PCB mass. An optimum mesh sized at 1800 three-dimensional cubic elements of length 1 (mm) was enough to have an absolute error of less than 1% compared to the analytical solution (Equation (17)).

#### 2.4.3. Boundary Conditions

The base of the L-35 cantilever model was fixed at the bottom surface by applying a surface fixed constrain, which included fixed displacement and rotation, as shown in Figure 6. The gyroscope mass is represented by a 5 (gm) point mass with a pinball region with a 5 (mm) radius. Thus, the gyroscope center is located 5 (mm) apart from the cantilever tip at the middle of the width. Nine input acceleration levels in the range from 0.1 to 2 (G) were applied to the cantilever model in 9 separate simulation runs to study the effect of input excitation.

#### 2.4.4. Simulation Details

In the beginning, a modal analysis of the L-35 cantilever model was conducted once under fixed geometrical boundary conditions to determine the natural frequency and select the proper frequency range for the harmonic analysis. After that, the harmonic analysis was conducted 9 times under a constant material density, elastic modulus and damping ratio, as well as the geometrical boundary conditions, to predict the frequency response of the L-35 jerk sensor. The nine individual simulation runs were performed under input accelerations of 0.1, 0.25, 0.5, 0.75, 1.0, 1.25, 1.5, 1.75 and 2.0 (G), respectively, whereas a variable frequency ranging from 0 to 300 (Hz) was applied in each run. For each input acceleration, the response spectrum (frequency response) and phase angle of the deflection, angle of deflection and angular velocity at the cantilever tip were predicted.

#### 2.4.5. Post-Processing of Data

From FE results, the output angular velocity at the L-35 cantilever tip ($\Omega_{tip}$) was predicted, and the acceleration sensitivity ($S_{a}$) was calculated by dividing the angular velocity ($\Omega_{tip}$) by the input acceleration ($a_{i}$). The jerk sensitivity ($S_{j}$) was obtained by dividing the acceleration sensitivity ($S_{a}$) by the input frequency ($\omega_{i}$) as in Equation (24). The input jerk ($j_{\left(t\right)}$) was calculated by dividing the angular velocity ($\Omega_{tip}$) by the jerk sensitivity ($S_{j}$) as in Equation (25).

As a result, the sensitivities of the L-35 jerk sensor to acceleration ($S_{a}$) ((deg/s)/(G)) and the jerk ($S_{j}$) ((deg/s)/(G/s)) were obtained as a function of frequency based on the angular velocity response spectrum predicted after each simulation run. To move from the mechanical domain (deg/s) to the electrical domain (mV), the output angular velocity ($\Omega_{tip}$) (deg/s) was multiplied by the sensitivity of the MPU9250 gyroscope suggested for measuring the angular velocity at the cantilever tip (MPU9250 has a sensitivity of 5 (mV/(deg/s)) at the measuring range of ±250 (deg/s)). As a result, the L-35 jerk sensor sensitivities $S_{a}$ and $S_{j}$ are expressed in the electrical domain in (mV/G) and (mV/(G/s)), respectively.

Based on the FE results and with the help of Equations (21), (24) and (25), the FE theoretical calibration curve of the L-35 jerk sensor, which relates the input jerk ($j_{\left(t\right)}$) with the gyroscope output volt (V) in the electromechanical domain, was then constructed for the seismic frequency range 0.1~40 (Hz), and its slope (sensitivity) was calculated.

In practical application, the experimental calibration curve is obtained by directly measuring the input acceleration ($a_{i}$) using the accelerometer mounted at the fixed end of the L-35 cantilever (as shown in Figure 1). At the same time, the angular velocity ($\Omega_{tip}$) is measured directly from the gyroscope mounted at the L-35 cantilever tip. A shaker is used to control the input frequency ($\omega_{i}$) and acceleration ($a_{i}$). It is worth noting that the accelerometer and the shaker are used only once for experimental calibration.

### 2.5. Experimental Work

The L-35 jerk sensor practical model was fabricated and calibrated using an electrodynamic shaker. A harmonic analysis was performed to obtain the experimental sensitivities and calibration curves.

#### 2.5.1. Sensor Fabrication

**Figure 7:**
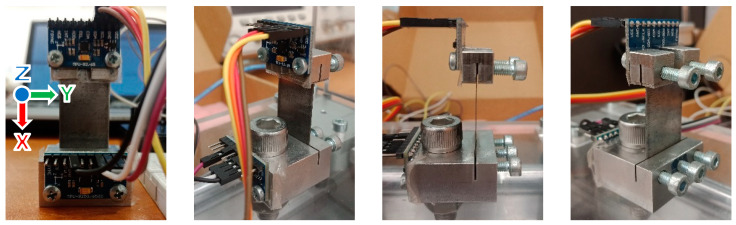
Photos of the fabricated L-35 jerk sensor from different angles.

The experimented L-35 jerk sensor consisted of four parts: a cantilever strip of dimensions 35 × 20 × 0.5 (mm^3^), a base of dimensions 30 × 30 × 15 (mm^3^), a gyroscope and an accelerometer of dimensions 25.5 × 15.4 × 1.5 (mm^3^), as shown in Figure 7. All body parts were made of austenitic spring stainless steel 304. The cantilever strip was fixed to the base inside a built-in grove with a thickness of 0.6 (mm) and depth of 5 (mm) using 3 M5 bolts. The fixation method was designed to facilitate the trial testing of different cantilevers. The mass of the gyroscope was 3 (gm). The gyroscope was installed on the cantilever where the z-axis direction was out of the plane, as shown in the left photo of Figure 7.

#### 2.5.2. Sensor Setup

Two 9-Axis MEMS sensors of type MPU9250 were attached to the L-35 jerk sensor, as shown in Figure 7. One was fixed at the cantilever free tip and was set up to output angular velocity data (i.e., gyroscope), while the other was fixed at the base and was set up to output acceleration data (i.e., accelerometer). MPU9250 can measure an angular velocity up to ±2000 (dps) with a sensitivity of 16.4 ± 3% (LSB/dps) and an acceleration up to ±16 (G) with a sensitivity of 2048 ± 3% (LSB/G). An Arduino Uno Rev 3.0 board was chosen as the data acquisition system (DAQ), which can read from 5 analogue channels and 14 digital channels at a baud rate up to 115,200 (bps). The gyroscope was configured to measure in the range of ±250 (dps) with a digital low-pass filter (DLPF) at a bandwidth of 92 (Hz) and an output delay of 3.9 (ms). The accelerometer was configured to measure in the range of ±2 (G) with a DLPF of 92 (Hz) and an output delay of 7.8 (ms). Arduino IDE V1.8.13 software running a customized C++ code was used to write the configurations and simultaneously read the gyroscope output in the y-axis and the accelerometer output in the z-axis. The Arduino code sampling frequency was 250 (Hz). We should emphasize that the accelerometer was used only once for determining the sensitivity curves $S_{a}$ and then $S_{j}$. In this manner, the jerk value was obtained directly from the experimental calibration curve.

#### 2.5.3. Experimental Work Details

**Figure 8:**
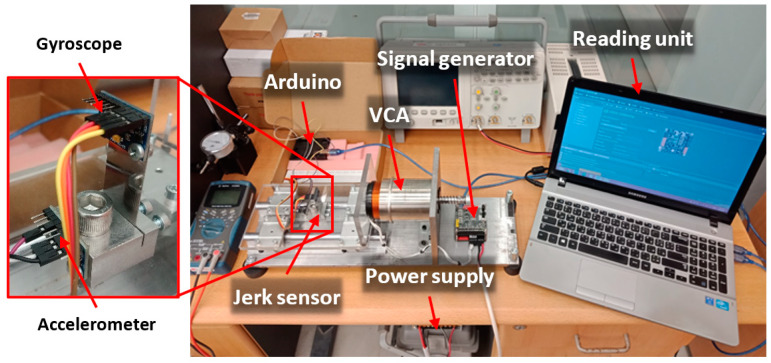
Photo of the high-excitation computer-controlled shaker for testing the L-35 jerk sensor and obtaining its calibration curve.

The experimental harmonic analysis of the L-35 jerk sensor was performed using a high-excitation computer-controlled electrodynamic shaker. The shaker consisted of a linear voice coil actuator (VCA), a uniaxial stage mounted on 4 roller bearings guided by 2 parallel rail guides, a DC servo drive controller with an I/O board and a power supply. A photo of the testing device is shown in Figure 8. The shaker can achieve a maximum mechanical frequency of 120 (Hz), a stroke distance of 25.4 (mm) and a continuous stall force of 185 (N) capable of generating an acceleration of 5 (G) at a payload mass of 3.5 (Kg). The mechanical frequency and amplitude were controlled using MotionLab V2.0 software installed on a laptop.

**Figure 9:**
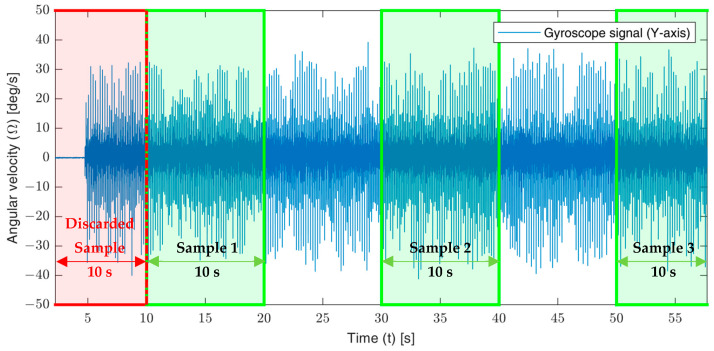
An illustration showing the extraction of the data samples from a recorded gyroscope signal.

The L-35 jerk sensor was subjected to 24 frequencies in the bandwidth of 5~120 (Hz). At each frequency, 5 accelerations were generated by altering the shaker stroke through adjusting the torque at 20, 40, 60, 80 and 100%. At each frequency and acceleration, the corresponding gyroscope signal was recorded using Arduino IDE V1.8.13 software for a time span of 60 (s). Three data samples of 10 s each were extracted from the total 60 s of the recorded signal at the start, middle and end as shown in Figure 9. Similarly, the accelerometer data-samples were extracted. The data samples were analyzed by the Fast Fourier Transformation (FFT) toolbox under MATLAB R2021a software to obtain the amplitude spectrum of the accelerometer and gyroscope signals in the frequency domain. The harmonic analysis results were used to construct the experimental sensitivity curves as previously discussed in Section 2.4.5.

For calibration, the jerk sensor was subjected to 40 input accelerations in the range of 0.1~1.2 (G). The input accelerations were generated at 20, 40, 60, 80 and 100% torque at each frequency of 5, 10, 15, 20, 25, 30, 35 and 40 (Hz). Then, the experimental calibration curve of the L-35 jerk sensor was constructed by plotting the output gyroscope voltage versus the input jerk for all the 40 input signals recorded in the frequency range of 5~40 (Hz).

## 3. Results

### 3.1. FE Results

#### 3.1.1. Modal Analysis

**Figure 10:**
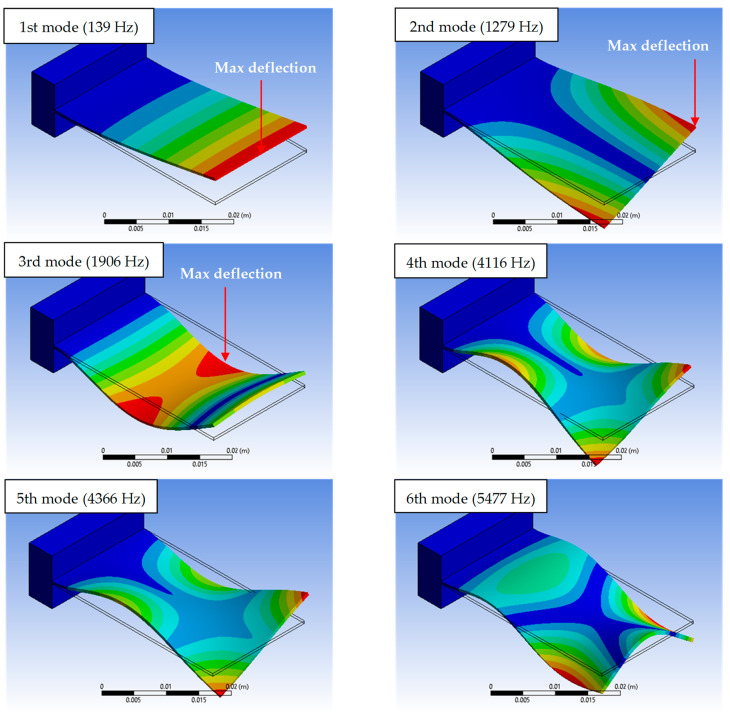
Natural mode shapes of the L-35 cantilever model.

Six modes of natural frequencies of the L-35 cantilever model are shown in Figure 10 as obtained from the modal analysis. This number of vibration modes is sufficient since the ratio between the sixth and first mode is about 40 times, whereas the ratio between the second and first mode is about 10 times. These ratios imply that the dominant mode of vibration is the first mode at 139 (Hz). This mode has been selected for studying the harmonic response since the deflection at the cantilever tip is a maximum that is very useful for the response of the angular velocity sensor at excitation frequencies below 139 (Hz). As shown in Figure 10, the shape mode at the first natural frequency is fairly linear. The second shape mode is due to torsion vibration, which is not recommended for the angular velocity sensor since the deflection at the midpoint of the tip is zero. The third mode is rejected since it is non-linear and maximum deflection occurs at the middle of the cantilever length. The fourth, fifth and sixth shape modes are complex and not recommended for working in the linear measurement zone. In addition, the first mode frequency is within 3~5 times the required range for the seismic vibration.

#### 3.1.2. Harmonic Analysis of the L-35 Cantilever

**Figure 11:**
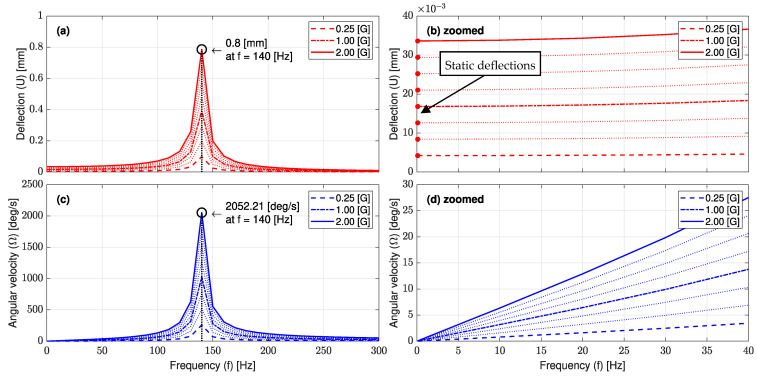
The combined effect of different magnitudes and frequencies of input acceleration on (**a**) the dynamic deflection with (**b**) a zoomed view into 0~40 (Hz), and (**c**) the angular velocity with (**d**) a zoomed view into 0~40 (Hz) at the tip of the L-35 stainless steel cantilever with damping ratio of 0.02.

The combined effect of different magnitudes and frequencies of input acceleration on dynamic deflection and angular velocity at the tip of the L-35 stainless steel cantilever is shown in Figure 11a,c, respectively. These results were obtained from the FE-damped harmonic response analysis under the acceleration range of 0.1~2 (G) and the frequency range of 0~300 (Hz), which included the first mode natural frequency, 139 (Hz). In the meantime, the damping ratio of the stainless steel cantilever material was taken as equal to 0.02 [35]. Regarding Figure 11a, it can be seen that for all input accelerations, the deflection increased slightly as the excitation frequency increased up to 110 (Hz), whereas it started to increase rapidly as the frequency approached the natural frequency of the L-35 cantilever. After that, peak values were achieved at 139 (Hz), whereas deflection drastically decreased again when the excitation frequency increased beyond 139 (Hz). At a 2 (G) input acceleration, the maximum elastic deflection value was 0.8 (mm), which was still within the elastic limit of the cantilever material. Angular velocity values at the cantilever tip showed similar trends under the same input frequency and acceleration conditions, as shown in Figure 11c. It is worthy to note that the angular velocity at the cantilever tip did not exceed 28 (deg/s) at 2 (G) and 40 (Hz), which is very suitable for commercial small-sized gyroscopes utilized for seismic measurement.

Harmonic deflections at 0 (Hz) have the same values as the static deflections, which ensure the validity of the harmonic model [36], and in turn, the angular velocity at the cantilever tip under different excitation accelerations can be accurately obtained. Table 3 lists deflection and angular velocity amplitudes at the tip of the L-35 cantilever as obtained from the FE harmonic analysis at 1, 40 and 139 (Hz) under different input accelerations. It is found that the angular velocity significantly changes with the input frequency, especially at the smallest amplitude. For instance, in Table 3 row 1, for a 0.1 (G) input acceleration generated at frequencies 1, 40 and 139 (Hz), the output angular velocity was 0.03, 1.38 and 102.61, respectively. However, at a 1 (Hz) constant input frequency with a variable input acceleration of 0.1, 0.25, 0.5, 0.75, 1, 1.25, 1.5, 1.75 and 2 (G), the measured angular velocity at the L-35 cantilever tip at 2 (G) reached 20 times the corresponding angular velocity value at 0.1 (G). This result confirms the great enhancement and high sensitivity of the designed L-35 cantilever.

#### 3.1.3. Theoretical Sensitivity of the L-35 Jerk Sensor

**Figure 12:**
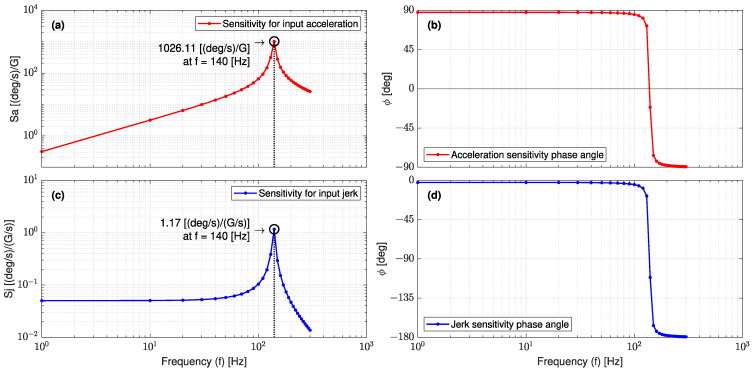
Bode diagrams as obtained from FEA for (**a**) sensitivity of jerk sensor for acceleration $\left|S_{a}\right|$; (**b**) $\left|S_{a}\right|$ phase angle; (**c**) sensitivity of jerk sensor for jerk $\left|S_{j}\right|$; and (**d**) $\left|S_{j}\right|$ phase angle.

In the log–log scale, Figure 12a,c show the predicted bode diagram of the jerk sensor sensitivity for input acceleration ($S_{a}$) and the jerk ($S_{j}$) in the frequency range of 0~300 (Hz), respectively. The peak value is observed at the natural frequency of 139 (Hz). From Figure 12a, it is found that the jerk sensor sensitivity for acceleration ($S_{a}$) is a linear function of frequency within the frequency range of 0.1~40 (Hz), and there is a 90 (deg) phase shift between the input and output up to 139 (Hz). The slope of the linear part represents the jerk sensor sensitivity for the input jerk ($S_{j}$), as denoted previously in Equation (24). Over the same range of frequency in Figure 12c, it is found that the jerk sensor sensitivity for the jerk ($S_{j}$) is a constant value of 0.052 ((deg/s)/(G/s)) and has a zero phase shift between the input jerk and output angular velocity. This implies that the jerk sensor reading ($j_{\left(t\right)}$) will be stable in the frequency range of 0.1~40 (Hz), which is very suitable for the desired seismic measurements. In addition, this sensitivity is almost five times the sensitivity value obtained by [26], which confirms the sensitivity enhancement of the L-35 cantilever-based jerk sensor.

**Figure 13:**
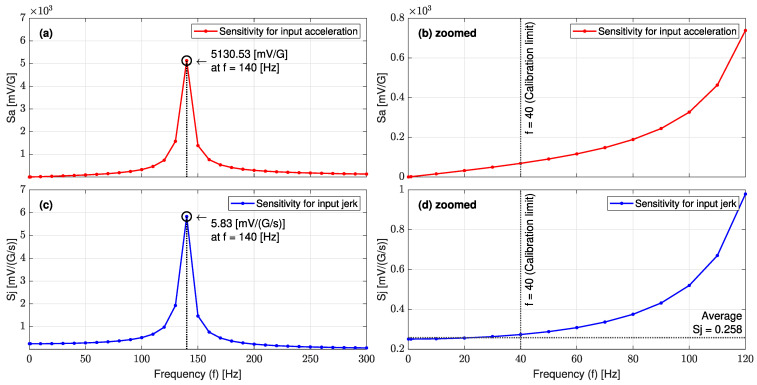
Jerk sensor sensitivity over the frequency range of 0~300 (Hz) for (**a**) input acceleration $\left|S_{a}\right|$ with (**b**) a zoomed view into 0~120 (Hz) and (**c**) input jerk $\left|S_{j}\right|$ with (**d**) a zoomed view into 0~120 (Hz).

In the electrical domain, Figure 13a,c illustrate the jerk sensor sensitivity for input acceleration ($S_{a}$) and the input jerk ($S_{j}$) over the frequency range of 0~300 (Hz), respectively. Figure 13b,d show zoomed views of the linear parts of $S_{a}$ and $S_{j}$. In the frequency range of 0.1~40 (Hz), it is seen that the jerk sensor sensitivity for the jerk ($S_{j}$) has a constant value of 0.258 (mV/(G/s)), whereas $S_{a}$ is a linear function of the input frequency. These trends of sensitivities are in very good agreement with the analytical equations (Equations (21) and (23)).

#### 3.1.4. Theoretical Calibration Curve of the L-35 Jerk Sensor

**Figure 14:**
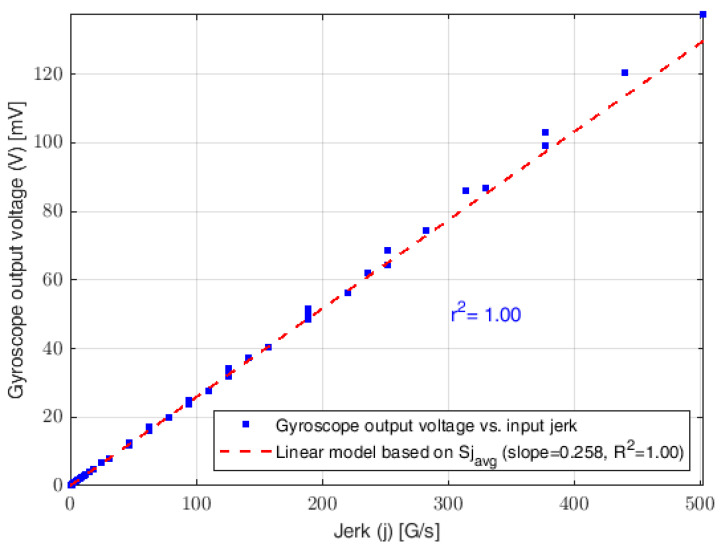
Theoretical calibration curve of the L-35 jerk sensor for input jerk at frequency range of 0.1~40 (Hz) and acceleration range of 0.1~2 (G) as predicted from the FE model.

Figure 14 illustrates the theoretical calibration curve of the L-35 jerk sensor for the input jerk at a frequency range of 0.1~40 (Hz) and an acceleration range of 0.1~2 (G). Thirty-six data points were fitted using a linear model based on $S_{j}$. The slope of the theoretical calibration curve, which represents the sensitivity of the L-35 jerk sensor model, is found to have an r-squared value of 0.99 and a ±1 linearity percentage. The theoretical maximum jerk of the L-35 model is found to be 4844 (G/s) or 47,512 (m/s^3^) at a 19 (G) input acceleration ($a_{i}$) and 40 (Hz) excitation frequency ($\omega_{i}$). The theoretical jerk limit is based on the gyroscope’s reference voltage limit of ±1250 (mV) at the measuring range of ±250 (deg/s).

### 3.2. Experimental Verification

#### 3.2.1. Experimental Harmonic Analysis

**Figure 15:**
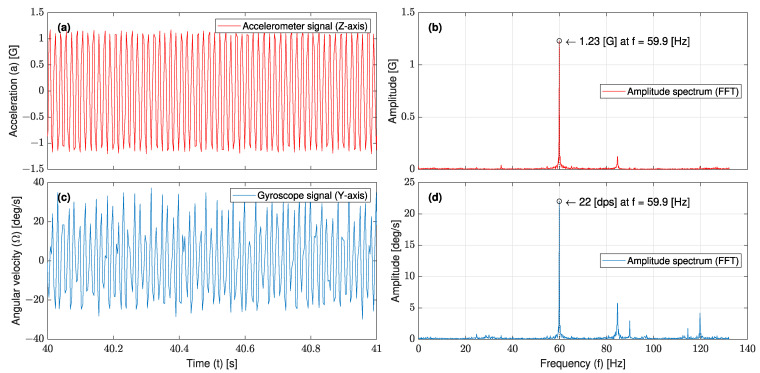
FFT analysis of a sample of the (**a**) accelerometer signal with its (**b**) amplitude spectrum, and (**c**) gyroscope signal with its (**d**) amplitude spectrum; signals were recorded at 60 (Hz) and 100% torque.

**Figure 16:**
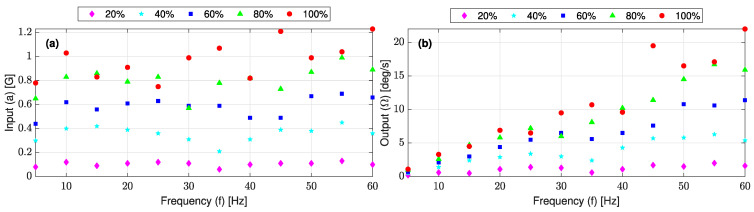
Maximum FFT peaks of (**a**) accelerometer and (**b**) gyroscope; signals were recorded in the frequency range of 5~60 (Hz).

Figure 15 shows the FFT analysis of a sample of the accelerometer and gyroscope signals recorded at 60 (Hz) and 100% torque. It is observed that while the dominant frequency is the input frequency, a few lower peaks appeared in the gyroscope signal spectrum and are attributed to some local frequencies of the shaker. Figure 16 shows the maximum FFT peaks of all the accelerometer and gyroscope signals recorded in the frequency range of 5~60 (Hz). The L-35 jerk sensor achieved the maximum output angular velocity response at the input frequency of 110 (Hz), about three times the required range for the seismic vibration. The experimental cantilever’s natural frequency is lower than that predicted by the FE modal analysis (139 (Hz)); this variation can be attributed to the presence of wires that affected the cantilever’s stiffness and was not considered during the simulation.

#### 3.2.2. Experimental Sensitivity Curves

**Figure 17:**
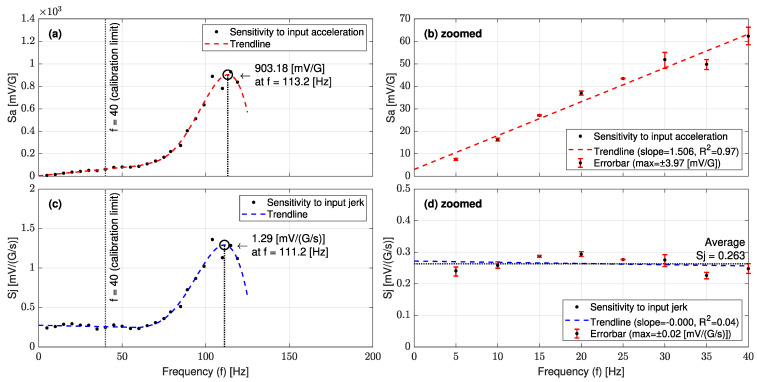
L-35 jerk sensor sensitivities for (**a**) input acceleration with (**b**) a zoomed view into 1~40 (Hz), and (**c**) input jerk with (**d**) a zoomed view into 1~40 (Hz).

Figure 17a,c show the experimental jerk sensor sensitivity curves for input acceleration ($S_{a}$) and the input jerk ($S_{j}$), respectively. From Figure 17d, the L-35 jerk sensor sensitivity for the input jerk ($S_{j}$) is almost constant in the range of 0.1~40 (Hz) with an average value of 0.263 (mV/(G/s)) or 0.053 ((deg/s)/(G/s)). This trend is in very good agreement with the theoretical sensitivity curve in Figure 13d. The validity test confirms that the enhanced L-35 cantilever-based jerk sensor is very efficient in sensing any variation in the input signal with a small amplitude.

#### 3.2.3. Experimental Calibration Curve

**Figure 18:**
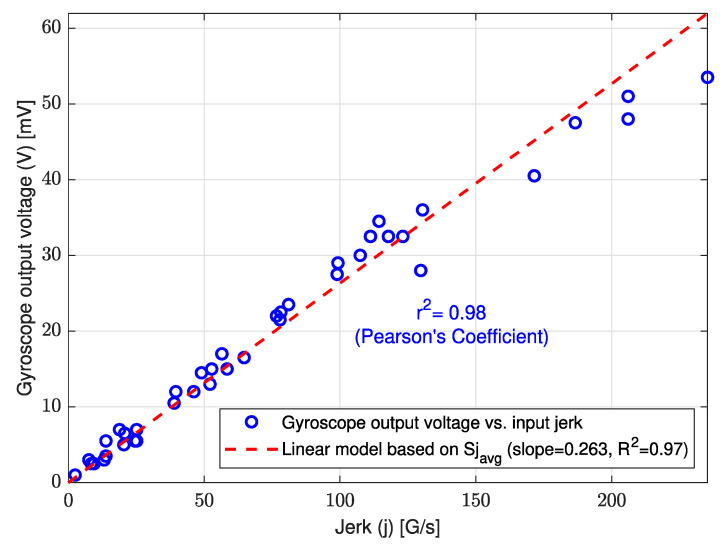
Experimental calibration curve of the L-35 jerk sensor for input jerk at frequency range of 0.1~40 (Hz) and acceleration range of 0.1~1.2 (G).

Figure 18 shows the experimental calibration curve of the L-35 jerk sensor for the input jerk at a frequency range of 0.1~40 (Hz) and an acceleration range of 0.1~1.2 (G). The linear fitting model is based on the average sensitivity for the input jerk and has a coefficient of determination ($R^{2}$) value of 0.97 with a high correlation coefficient ($r^{2}$) value of 0.98.

### 3.3. Performance Comparison of the L-35 Jerk Sensor

Table 4 summarizes the performance of the L-35, A [26], B [26] and JW-1 [21] jerk sensors in terms of the sensitivity, bandwidth, measurable range and linearity percentages. The enhanced L-35 jerk model gives a sensitivity 5 times higher than model A and 13 times higher than model B. The measurable jerk range of L-35 is five times wider than that of model JW-1, three times wider than model B and two times wider than model A. The linearity percentage is identical for all models and is ±1%. This comparison ensures the remarkable enhancement in the measurement performance of the L-35 cantilever-based jerk sensor presented in this study.

## 4. Summary and Conclusions

In this study, a cantilever-based sensor was proposed for the direct measurement of the jerk. The jerk sensor was analytically modeled, simulated by FEA and experimentally verified to enhance its measurement performance in terms of sensitivity and the jerk measurable range in the seismic frequency bandwidth. The sensor structure consists of an austenitic stainless steel 304 cantilever and an angular velocity meter (gyroscope) mounted on the cantilever tip. The L-35 cantilever of dimensions 35 × 20 × 0.5 (mm^3^) was selected and fabricated to work in the bandwidth of seismic measurements (0.1~40 (Hz)), and its natural frequency was experimentally found to be 111 (Hz). Analytical and FE analyses of the L-35 jerk sensor were performed to predict the theoretical sensitivity ($S_{j}$) and calibration ($j_{\left(t\right)}$) curves. The L-35 was tested on a high excitation computer-controlled shaker to obtain the experimental sensitivity and calibration curves. The jerk sensor’s theoretical and experimental calibration curves were found to be linear in the bandwidth of 0.1~40 (Hz) with a linearity r-squared value of 0.99 and 0.98, respectively. The experimental and theoretical jerk sensitivity ($S_{j}$) was found to be in excellent agreement with a ±2% error and had a constant value of 0.05 ((deg/s)/(G/s)) for the excitation frequencies below 40 (Hz). The sensitivity of the enhanced jerk sensor has reached five times the sensitivity of the cantilever-based jerk sensor reported in [26], with a wider jerk measurable range of 47,000 (m/s^3^). The sensor can be utilized in other applications that require direct measurement of the force rate of change such as detection of discontinuities in signals during low cyclic fatigue testing.

## Figures and Tables

**Table 1:** First mode natural frequency of 18 cantilever dimension sets as estimated from Equation (26) and modal FEA.

Model	Length (L) (mm)	Width (w) (mm)	Thickness (t) (mm)	First Mode Natural Frequency $\left(f_{n}\right)$ (Hz)	
				Equation (26)	FEA
1	30	15	0.5	166.55	162.21
2			1	419.01	425.81
3			1.5	700.13	724.35
4		20	0.5	184.37	183.61
5			1	453.30	474.7
6			1.5	746.52	798.0
7	35	15	0.5	127.27	122.82
8			1	316.34	321.95
9			1.5	524.49	548.05
10 * (L-35)		20	0.5	140.23	138.95
11			1	340.35	358.44
12			1.5	556.22	602.68
13	40	15	0.5	100.90	96.932
14			1	248.06	253.52
15			1.5	408.52	431.38
16		20	0.5	110.69	109.56
17			1	265.61	281.76
18			1.5	431.27	473.34

* Dimensions set that meet the design criteria (3~5 times 40 (Hz)).

**Table 2:** Mechanical properties of L-35 stainless steel cantilever.

Property	Value	Unit
Density	7969	(kg/m^3^)
Young’s Modulus	195	(GPa)
Poisson’s Ratio	0.27	
Bulk Modulus	141.3	(GPa)
Shear Modulus	76.772	(GPa)
Tensile Yield Strength	252.1	(MPa)
Tensile Ultimate Strength	565.1	(MPa)
Damping Ratio [35]	0.02	
Constant Structural Damping Coefficient [35]	0.04	(kg/m^3^)

**Table 3:** Deflection $\left(U_{tip}\right)$ and angular velocity $\left(\Omega_{tip}\right)$ amplitudes at the tip of the L-35 cantilever as obtained from FE harmonic analysis at 1, 40 and 139 (Hz) under different input accelerations.

**Input Acceleration** **(G)**	**1 (Hz)**		**40 (Hz)**		**139 (Hz)**	
	$U_{tip}$ (mm)	$\Omega_{tip}$ (deg/s)	$U_{tip}$ (mm)	$\Omega_{tip}$ (deg/s)	$U_{tip}$ (mm)	$\Omega_{tip}$ (deg/s)
0.10	0.002	0.03	0.002	1.38	0.039	102.61
0.25	0.004	0.08	0.005	3.44	0.098	256.53
0.50	0.008	0.16	0.009	6.88	0.196	513.05
0.75	0.013	0.24	0.014	10.32	0.295	769.58
1.00	0.017	0.32	0.018	13.76	0.393	1026.11
1.25	0.021	0.39	0.023	17.21	0.491	1282.63
1.50	0.025	0.47	0.027	20.65	0.589	1539.6
1.75	0.029	0.55	0.032	24.09	0.687	1795.69
2.00	0.034	0.63	0.037	27.53	0.789	2052.21

**Table 4:** Comparison between performance of L-35, A, B and JW-1 jerk sensor models.

**Property**	**L-35**	A *	B *	JW-1 **
Dimensions (mm)	35 × 5 × 0.5	145 × 30 × 5	20 × 30 × 1.2	N/A
Material	Stainless steel 304	Aluminum	Aluminum	N/A
Natural frequency (Hz)	111	90	160	N/A
Sensitivity ((deg/s)/(G/s))	0.053	0.01	0.004	N/A
Gyroscope sensitivity (mV/(deg/s))	5	25	25	N/A
Sensitivity (mV/(m/s^3^))	0.03	0.02	0.01	0.08
Bandwidth	0.1–40	0.1–60	0.1–60	0.3–100
Measurable Range (m/s^3^)	47,000	14,000	35,000	10,000
Linearity (%)	±1	±1	±1	±1

* Models A and B by Tamura et al. [26]; ** Model JW-1 by Xueshan et al. [21].

## Data Availability

Some or all data, models or code that support the findings of this study are available from the corresponding author upon reasonable request. The data list includes FEA models, FEA results, post-processing calculation sheets, MEMS component data sheets, Arduino codes, MATLAB codes and any detailed engineering drawings.
